# Tilt Effects in Optical Angle Measurements

**DOI:** 10.6028/jres.099.057

**Published:** 1994

**Authors:** Yun H. Queen

**Affiliations:** National Institute of Standards and Technology, Gaithersburg, MD 20899-0001

**Keywords:** AAMACS, angle blocks, angles, autocollimator, calibrations, polygons, tilt

## Abstract

Vector analysis is used to determine the quantitative error in angle calibration using autoeollimators. This error is caused by tilt in the mount upon which the artifact is placed. For tilt angles that are less than 1°, the error can be simplified to be the product of a eoeffieient and three terms. The three terms are: (1) the square of the tilt, (2) the sine of the artifact’s nominal angle, and (3) the eosine of the artifact’s nominal angle plus two times the artifact’s position angle. It is shown that the error can be eliminated by placing the artifact at designated periodic positions.

## 1. Introduction

In 1990, the National Institute of Standards and Technology (NIST) acquired a new angle calibration instrument, the Advanced Automated Master Angle Calibration System (AAMACS), consisting of three stacked indexing tables. The AAMACS indexes to any angle position with a resolution of 0.003 arcsecond. Furthermore, the repeatability for each table is within ± 0.03 arcsecond. Because of the acquisition of this highly repeatable and highly accurate instrument, the angle calibration service must redefine its procedures to minimize errors that were previously ignored. Six of these error sources are: (1) tilt in the mount, (2) seismic or acoustic vibration, (3) thermal drift and distortion, (4) air turbulence and refraction, (5) non-flatness of the artifact mirrors, and (6) autocollimator errors, including optical distortions, axis cross-talk, and calibration error. Of these error sources, only the tilt effect is discussed in this paper.

Errors due to the tilt effect in angle measurements have long been known. However, quantitative information about the tilt effect was unclear. Traditionally, the error in the measurement of angle blocks was averaged out with two measurements. The first measurement was done with the top of the artifact in an upward position and the second measurement was done with the bottom of the artifact in an upward position. This process did not reduce the error, but introduced more error if the top and bottom surfaces were not parallel.

The tilt effect was noted in the 1960’s when Hume [1], using an autocollimator, measured an optical polygon placed on a tilted mount. By rotating the mount, the autocollimator’s elevation or vertical reading changed direction. As a result, Hume recommended that the tilt must not be more than 2 arcminutes during a calibration. However, no quantitative indication of the error incurred was given Therefore, the intention of this paper is to show how much the error of angle measurements results from tilt.

## 2. Instrumentation and Setup

An autocollimator was used to measure the angle position of a flat surface by directing a beam of light toward the surface. The light reflected back into the autocollimator was detected by a photocell, photomultiplier tube, or a CCD array. Inside the autocollimator, the angle of reflection was compared to a reference angle which was either single axis of dual axis [2]. The measuring axis was required to be squarely horizontal or vertical with respect to the axis of rotation.

**Fig. 1:**
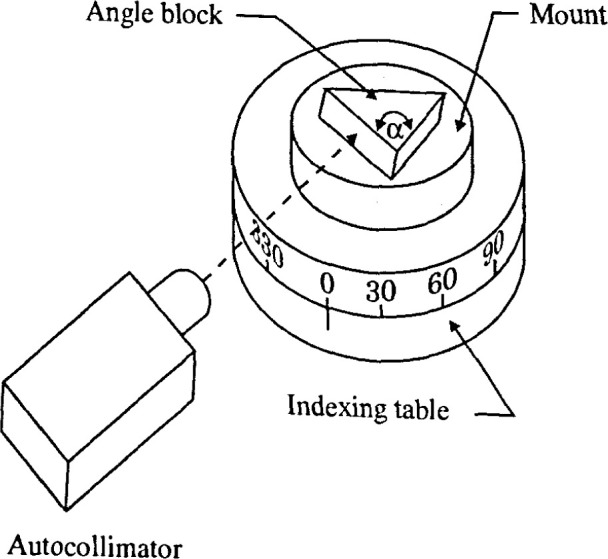
Angle block calibration setup with no tilt in the mount.

**Fig. 2:**
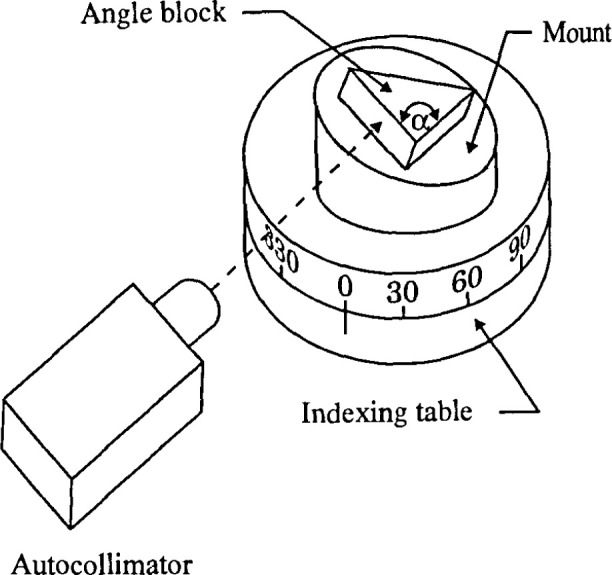
Angle block calibration setup with tilt in the mount.

For the analysis, an angle block was used as the artifact. The purpose of the calibration was to determine the corner angle formed between the two faces of the angle block. Although a nominal angle value, *α*, was provided by the manufacturer, this value may not be the true angle, thus requiring calibration. The following example shows a typical setup for an angle block calibration [3]. In Fig. 1, the angle block was wrung on top of a mount placed on an indexing table. One surface of the angle block was aligned with the autocollimator and its azimuth or horizontal position measured. The indexing table was then rotated to (180° − *α*) from the initial position where the other surface of the angle block was measured. Assuming the indexing table is perfect, the difference between the two angle measurements gives the true angle value from the nominal value. Figure 2 shows the same setup as Fig. 1 except that the mount is tilted with respect to the axis of rotation. Figure 2 measurements are different from those of Fig. 1 because of the presence of the tilt. This difference is the error in the angle measurement.

## 3. Theory

**Fig. 3:**
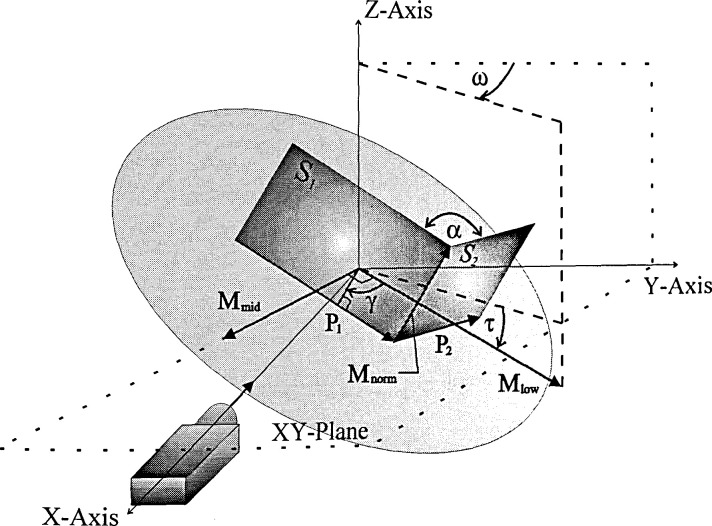
Angle block calibration setup represented by vectors.

Figure 3 illustrates the setup of Fig. 2 with appropriate vectors. Three assumptions were made for the analysis: (1) the nominal angle was the true value, (2) the indexing table was perfect, and (3) the angle block was perpendicularly mounted on top of the mount. All three assumptions were justified because the actual values could be determined with a sufficiently small uncertainty.

### 3.1 Spatial Definitions

The coordinate space is defined by a right handed *xyz*-coordinate system. Its origin is in the center of the mount and the axis of rotation is in the *z*-axis. Four independent variables were considered: (1) the nominal angle, *α*, of the angle block, (2) the tilt angle, *τ*, of the mount, (3) the position angle, *γ*, of the angle block on the mount, and (4) the horizontal angle, *ω*, of the mount. The first independent variable was discussed above. The second variable, *τ*, is the smallest angle between *M*_low_ (vector from the origin to the lowest point of the mount) and the *xy*-plane. The third variable, *γ*, is the angle between the normal of the artifact’s first surface, *S*_1_, and *M*_low_. The last variable, *ω*, is the angle between the *M*_low_*z*-plane and the *yz*-plane.

Initially, the angle block was placed as close as possible to the center of the mount where surface *S*_1_ was aligned with the autocollimator which was placed concentrically with the *x*-axis. The autocollimator light source was in the −*i* direction. Consequently, the reflected light was in the *i* direction. The horizontal angle of the surface measured by the autocollimator was in the positive *x*-axis half of the *xy*-plane. This angle is measured positive in the clockwise direction from the *x*-axis as viewed from the positive *z*-axis.

### 3.2 Angle Definitions

There are 360 degrees (°) in a circle, 60 arcminutes (′) in a degree, and 60 arcseconds (″) in an arcminute. Therefore, there are 3600 arcseconds in a degree.

### 3.3 Analysis

Step 1. The normal vector of the mount, *M*_norm_, is obtained by taking the cross product of *M*_mid_ and *M*_low_. The vector *M*_mid_ is on the mount at 90° clockwise from *M*_low_. These unit vectors are dependent on *τ* and *ω* as follows:

$$M_{\text{norm}}=M_{\text{mid}}\times M_{\text{low}}$$ (1)

$$M_{\text{low}}=\cos\left(\tau \right)\sin\left(\omega \right)i+\cos\left(\tau \right)\cos\left(\omega \right)j-\sin\left(\tau \right)k$$ (2)

$$M_{\text{mid}}=\cos\left(\omega \right)i-\sin\left(\omega \right)j$$ (3)

where *τ* is less than 90° and *ω* is between 0° and 360°, inclusive. These two angles are obtained experimentally.

Step 2. The vector, *P*_1_, is related to the mount vectors by the following dot products:

$$P_{1}\cdot M_{\text{low}}=\cos\left(\theta_{1}\right)$$ (4)

$$P_{1}\cdot M_{\text{mid}}=\cos\left(\theta_{2}\right)$$ (5)

$$P_{1}\cdot M_{\text{norm}}=\cos\left(\theta_{3}\right)$$ (6)

where

$$\theta_{1}=\gamma -90{}^{\circ}$$ (7)

$$\theta_{2}=\theta_{1}-90{}^{\circ}$$ (8)

$$\theta_{3}=90{}^{\circ}.$$ (9)

The three components of *P*_1_ are determined by solving the above three simultaneous equations.

Step 3. The normal vector to surface *S*_1_, *N*_1_, is obtained by taking the cross product of *P*_1_ and *M*_norm_ as follows:

$$N_{1}=P_{1}\times M_{\text{norm}}.$$ (10)

Step 4. The horizontal angle of surface *S*_1_ measured by the autocollimator is the projection of *N*_1_ onto the *xy*-plane:

$$\theta_{H:S_{1}}=\arcsin\left(-\frac{N_{1y}}{\sqrt{N_{1x}^{2}+N_{1y}^{2}}}\right).$$ (11)

At this point, I would like to rectify an error in a previous paper [4] on the same topic. Equation (16) written as *κ* = l + sin^2^(*τ*), should be *κ* = l + tan^2^(*τ*). If the corrected equation is substituted into Eqs. (14) and (15) of the previous paper these equations would be the same as the above Eqs. (13) and (14), respectively.

## 4. Theoretical Results

During calibration, it is customary to align the first surface of the artifact to the reference zero of the autocollimator as much as possible. Therefore, the mount angle, *ω*, during setup is determined by the position of the artifact (see Appendix A). As a result, the horizontal angle error is a function of just *α*, *τ*, and *γ*.

**Fig. 4:**
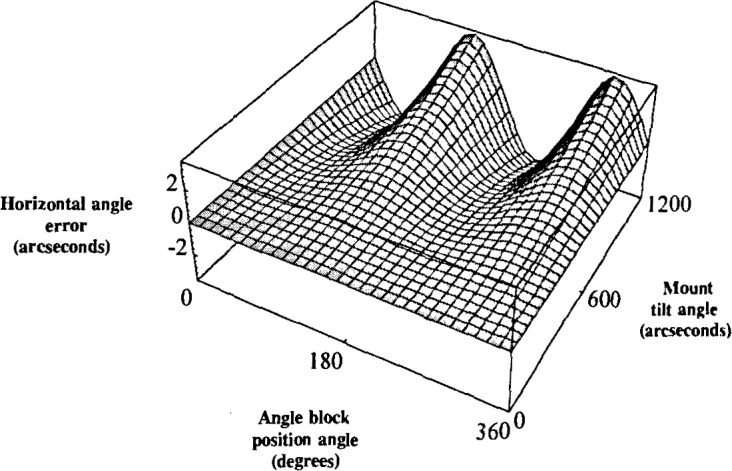
Theoretical plot of the horizontal angle error for a 90° angle block with respect to the tilt angle and the artifact position angle.

This error is plotted in Fig. 4 for a 90° angle block with respect to the tilt and block position. The tilt angle ranged from 0″ to 1200″ and the position angle ranged from 0° to 360°. As anticipated, the magnitude of the error increased with increased tilt. Note that despite an increase in tilt, when the artifact’s position angle is 0°, 90°, 180°, or 270°, the error is zero.

Step 5. The horizontal angle for surface *S*_2_ is similarly obtained. First, the mount is rotated to a position such that surface *S*_2_ is aligned with the autocollimator. Thus, the new mount angle becomes (180° − *α*) + *ω*_(old)_. The tilt angle, the nominal angle, and the artifact’s position angle remain unchanged. The angle between *P*_2_ and *M*_low_ becomes − (180° − *α*) + *θ*_1(old)_. The first four steps are repeated to solve for the horizontal angle of surface *S*_2_. The differences in these horizontal angles, *ϵ*_H_, is the error of angle measurement resulting from the tilt.

Step 6. The horizontal angle error, *ϵ*_H_, is a function of *τ*, *γ*, *α*, and *ω*:

$$\epsilon_{H}=\theta_{H:S_{2}}-\theta_{H:S_{1}}$$ (12)

where

$$\theta_{H:S_{1}}=-\arctan\left(\frac{\cos\left(\tau \right)\cos\left(\gamma \right)\cos\left(\omega \right)-\sin\left(\gamma \right)\sin\left(\omega \right)}{\cos\left(\tau \right)\cos\left(\gamma \right)\sin\left(\omega \right)+\sin\left(\gamma \right)\cos\left(\omega \right)}\right)$$ (13)

$$\theta_{H:S_{2}}=-\arctan\left(\frac{\cos\left(\tau \right)\cos\left(\alpha +\gamma \right)\cos\left(\omega +108{}^{\circ}-\alpha \right)-\sin\left(\alpha +\gamma \right)\sin\left(\omega +108{}^{\circ}-\alpha \right)}{\cos\left(\tau \right)\cos\left(\alpha +\gamma \right)\sin\left(\omega +108{}^{\circ}-\alpha \right)-\sin\left(\alpha +\gamma \right)\cos\left(\omega +108{}^{\circ}-\alpha \right)}\right).$$ (14)

Intuitively, this is expected. If surface *S*_1_ is placed right at or right along the lowest tilt, surface *S*_2_ by default is right along or right at the lowest tilt, respectively. Consequently, only the vertical angle is changed. However, when the position of the block is not placed at those locations, the error increases with an increase in tilt. This shows that by placing the artifact at strategic points, the error is eliminated.

**Fig 5:**
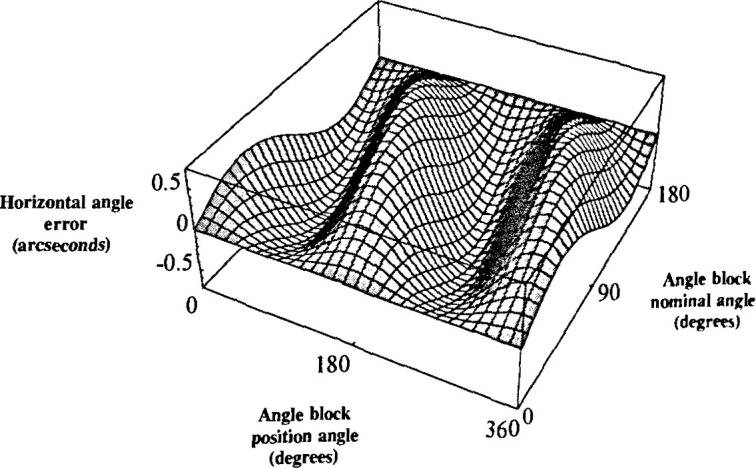
Theoretical plot of the horizontal angle error for a tilt of 9′ with respect to the artifact nominal angle and position angle.

Figure 5 shows the horizontal angle error for a 9′ tilt angle with respect to the nominal and block position. The nominal angle ranged from 0° to 180° and the position angle ranged from 0° to 360°. As anticipated, the horizontal angle error is bounded when the tilt angle is fixed.

## 5. Simplification of the Horizontal Angle Error

The horizontal angle error may be simplified, using a Taylor-series expansion:

$$\epsilon_{H}=\left(0.5\right)\tau^{2}\sin\left(\alpha \right)\cos\left(\alpha +2\gamma \right)+O_{1}\left(\alpha ,\gamma ,\tau \right)$$ (15)

$$\epsilon_{H}=\left(2.424\times 10^{-6}\right)\tau^{2}\sin\left(\alpha \right)\cos\left(\alpha +2\gamma \right)+O_{2}\left(\alpha ,\gamma ,\tau \right)$$ (16)

where *ϵ*_H_ and *τ* are expressed in radians in Eq. (15) and in arcseconds in Eq. (16). The terms *O*_1_(*α*, *γ*, *τ*) and *O*_2_(*α*, *γ*, *τ*) represent the sum of the remaining terms that are negligible for tilt angles less than 1° (see Appendix A).

The above equations show that the horizontal angle error is zero if the cosine term equals zero. This occurs when the artifact position, *γ*, is

$$\gamma =45{}^{\circ}-\frac{\alpha }{2}\pm 90{}^{\circ}N$$ (17)

where *N* is a whole number from 0 to ∞. This means that if a 90° block is placed at 0°, 90°, 180°, or 270°, and so on al every 90° increment, the error is zero.

For constant tilt angles, the above equations show that the error is bounded by the maximum amplitude, $A_{\epsilon_{H}}$:

$$A_{\epsilon_{H}}=\left(0.5\right)\tau^{2}$$ (18)

$$A_{\epsilon_{H}}=\left(2.424\times 10^{-6}\right)\tau^{2}$$ (19)

where $A_{\epsilon_{H}}$ and *τ* are expressed in radians in Eq. (18) and in arcseconds in Eq. (19).

## 6. Results

**Fig. 6:**
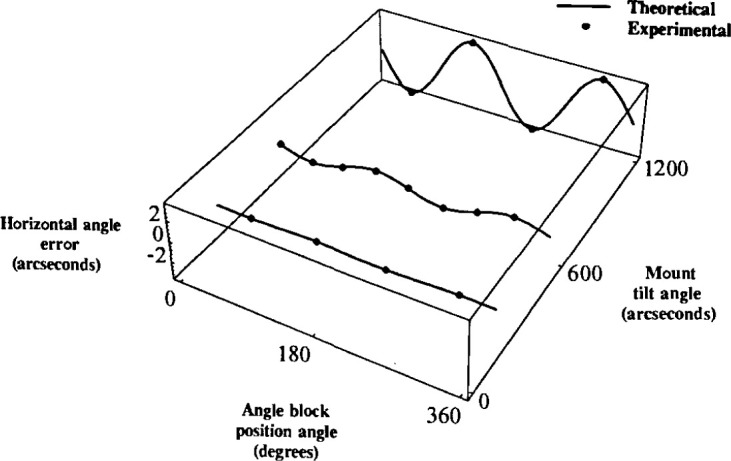
Theoretical and experimental comparison of the horizontal angle error for a 90° angle block at tilt angles of 3′, 9′, and 20′ as a function of position angle.

An experiment was performed which determined the horizontal angle error of a 90° angle block placed on mounts with tilt angles of 3′, 9′, and 20′. In all cases, the block was placed at the maximum tilt effect positions. For the 9′ case, the block was also placed at the zero tilt effect positions. Experimental results were compared with the theoretical results. The experimental results agreed very well with the predicted results with a difference of 0.04″ being the worst case. Note the similarity between Fig. 4 and Fig. 6.

**Fig. 7:**
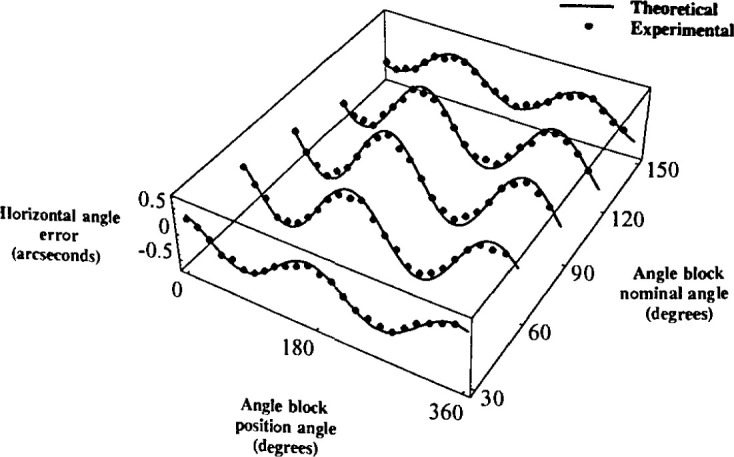
Theoretical and experimental comparison of the horizontal angel error for 30°, 60°, 90°, 120°, 150° angel blocks, all at 9′ tilt as a function of position angle.

A similar experiment was performed in which angle blocks of 30°, 60°, 90°, 120°, and 150° were placed on an approximately 9′ tilt mount. In this experiment, the angle blocks were placed at twenty-four evenly spaced positions from the lowest point of the tilt. Again, experimental results were compared with the predicted results, and again, they agreed well; the largest difference was only 0.17″. Note the similarity between Fig. 5 and Fig. 7.

For both experiments, each point was obtained by taking the average of two measurements. The first measurement was obtained by the conditions described. The second measurement was obtained by placing the angle block at 180° from its prescribed position. This was done for two reasons. First, in practical experience, there was a high probability of measurement error caused by the non-flatness of the measured surfaces and by the distortion in the autocollimator optics. This meant that the autocollimator readings varied with the shifted positions of a surface even if the angle position of that surface remained the same. As a result, the measurement must be compensated by shifting the surface in the opposite direction. This was accomplished by placing the artifact at 180° from its original position. Second, since the predicted curve repeated itself at every 180°, measuring the block at 180° from the original position yielded the same result as if the block were at the original position. This averaging method was useful since it resulted in good agreement between the measured and the predicted values.

Note also that the results of the first experiment as shown in Fig. 6 were overall better than those of the second as shown in Fig. 7. This was probably due to the better surface flatness of the artifact used in the experiment for Fig. 6 than that for Fig. 7.

## 7. Summary

A formula was developed which gives the quantitative error in optical angle measurements due to the presence of tilt. This formula, derived using vector analysis, shows that the error is a function of the tilt angle, the nominal angle, and the position angle of the artifact. For tilt angles that are less than 1°, the error can be simplified as the product of a coefficient and three terms. The three terms are: (1) the square of the tilt, (2) the sine of the artifact’s nominal angle, and (3) the cosine of the artifact’s nominal angle plus two times the artifact’s position angle. It is shown that the error can be eliminated by placing the artifact at designated periodic positions.

Two experiments were performed to verify this formula. The first experiment measured the horizontal angle error of a 90° angle block at tilt angles of 3′, 9′, and 20′ as a function of block position. The second experiment measured the horizontal angle error of 30°, 60°, 90°, 120°, and 150° angle blocks with about 9′ of tilt as a function of block position. Remarkable agreement between the predicted and the experimental results was found; the largest difference observed was 0.17″. This good agreement was obtained even with large tilt angles. The formula may also be used for optical polygons to determine the tilt error for each of its adjacent surfaces.

## 8. Appendix A. Derivation of Horizontal Angle Error in Angle Block Measurements

### 8.1 Derivation of the Horizontal Angle Position for Surface 1

Initially, the mount has a tilt *τ* and is at position *ω*. Therefore,

$$\begin{array}{c} M_{\text{notm}}=M_{\text{mid}}\times M_{\text{low}}\\ M_{\text{low}}=\cos\left(\tau \right)\sin\left(\omega \right)i+\cos\left(\tau \right)\cos\left(\omega \right)j-\sin\left(\tau \right)k\\ M_{\text{mid}}=\cos\left(\omega \right)i-\sin\left(\omega \right)j \end{array}$$

which gives

$$M_{\text{norm}}=\sin\left(\tau \right)\sin\left(\omega \right)i+\cos\left(\omega \right)\sin\left(\tau \right)j+\cos\left(\tau \right)k.$$

Next, we have

$$\begin{array}{c} P_{1}\cdot M_{\text{low}}=\cos\left(\theta_{1}\right)\\ P_{1}\cdot M_{\mathrm{mid}}=\cos\left(\theta_{2}\right)\\ P_{1}\cdot M_{\mathrm{norm}}=\cos\left(\theta_{3}\right) \end{array}$$

where

$$\begin{array}{c} \theta_{1}=\gamma -90{}^{\circ}\\ \theta_{2}=\theta_{1}-90{}^{\circ}\\ \theta_{3}=90{}^{\circ} \end{array}$$

which gives

$$\begin{array}{c} \left[\begin{array}{ccc} \cos\left(\tau \right)\sin\left(\omega \right) & \cos\left(\tau \right)\cos\left(\omega \right) & -\sin\left(\tau \right)\\ \cos\left(\omega \right) & -\sin\left(\omega \right) & 0\\ \cos\left(\tau \right)\sin\left(\omega \right) & \cos\left(\omega \right)\sin\left(\tau \right) & \cos\left(\tau \right) \end{array}\right]\left[\begin{array}{c} P_{1x}\\ P_{1y}\\ P_{1z} \end{array}\right]\\ =\left[\begin{array}{c} \cos\left(\gamma -90{}^{\circ}\right)\\ \cos\left(\left(\gamma -90{}^{\circ}\right)-90{}^{\circ}\right)\\ \cos\left(90{}^{\circ}\right) \end{array}\right]. \end{array}$$

Solving for *P*_1_ in the above matrix yields its three components:

$$\left[\begin{array}{c} P_{1x}\\ P_{1y}\\ P_{1z} \end{array}\right]=\left[\begin{array}{c} -\cos\left(\gamma \right)\cos\left(\omega \right)+\cos\left(\tau \right)\sin\left(\gamma \right)\sin\left(\omega \right)\\ \cos\left(\tau \right)\cos\left(\omega \right)\sin\left(\gamma \right)+\cos\left(\gamma \right)\sin\left(\omega \right)\\ -\sin\left(\gamma \right)\sin\left(\tau \right) \end{array}\right].$$

Next, we solve

$$N_{1}=P_{1}\times M_{\text{norm}}$$

which gives the three components of *N*_1_:

$$\left[\begin{array}{c} N_{1x}\\ N_{1y}\\ N_{1z} \end{array}\right]=\left[\begin{array}{c} \cos^{2}\left(\tau \right)\cos\left(\omega \right)\sin\left(\gamma \right)+\cos\left(\omega \right)\sin\left(\gamma \right)\sin^{2}\left(\tau \right)+\cos\left(\gamma \right)\cos\left(\tau \right)\sin\left(\omega \right)\\ \cos\left(\gamma \right)\cos\left(\tau \right)\cos\left(\omega \right)-\cos^{2}\left(\tau \right)\sin\left(\gamma \right)\sin\left(\omega \right)-\sin\left(\gamma \right)\sin^{2}\left(\tau \right)\sin\left(\omega \right)\\ -\cos\left(\gamma \right)\sin\left(\tau \right) \end{array}\right].$$

Further, we have

$$\theta_{H:S_{1}}=\arcsin\left(-\frac{N_{1y}}{\sqrt{N_{1x}^{2}+N_{1y}^{2}}}\right)$$

which is equivalent to

$$\theta_{H:S_{1}}=-\arctan\left(\frac{N_{1y}}{N_{1x}}\right).$$

Therefore,

$$\theta_{H:S_{1}}=-\arctan\left(\frac{\cos\left(\tau \right)\cos\left(\gamma \right)\cos\left(\omega \right)-\sin\left(\gamma \right)\sin\left(\omega \right)}{\cos\left(\tau \right)\cos\left(\gamma \right)\sin\left(\omega \right)+\sin\left(\gamma \right)\cos\left(\omega \right)}\right).$$

### 8.2 Derivation of the Horizontal Angle Position for Surface 2

The mount is rotated to *ω*_(new)_ such that surface *S*_2_ is in alignment with the autocollimator as:

$$\omega_{\left(\text{new}\right)}=\omega +\left(180{}^{\circ}-\alpha \right).$$

Therefore,

$$\begin{array}{c} M_{\text{low}}=\cos\left(\tau \right)\sin\left(\omega +180{}^{\circ}-\alpha \right)i+\cos\left(\tau \right)\cos\left(\omega +180{}^{\circ}-\alpha \right)j-\sin\left(\tau \right)k\\ M_{\text{mid}}=\cos\left(\omega +180{}^{\circ}-\alpha \right)i-\sin\left(\omega +180{}^{\circ}-\alpha \right)j\\ M_{\text{norm}}=\sin\left(\tau \right)\sin\left(\omega +180{}^{\circ}-\alpha \right)i+\cos\left(\omega +180{}^{\circ}-\alpha \right)\sin\left(\tau \right)j+\cos\left(\tau \right)k. \end{array}$$

Also for surface 2,

$$\theta_{1\left(\text{new}\right)}=\theta_{1\left(\text{old}\right)}-\left(180{}^{\circ}-\alpha \right).$$

As a result, the matrix for solving *P*_2_ becomes

$$\left[\begin{array}{ccc} \cos\left(\tau \right)\sin\left(\omega +180{}^{\circ}-\alpha \right) & \cos\left(\tau \right)\cos\left(\omega +180{}^{\circ}-\alpha \right) & -\sin\left(\tau \right)\\ \cos\left(\omega +180{}^{\circ}-\alpha \right) & -\sin\left(\omega +180{}^{\circ}-\alpha \right) & 0\\ \sin\left(\tau \right)\sin\left(\omega +180{}^{\circ}-\alpha \right) & \cos\left(\omega +180{}^{\circ}-\alpha \right)\sin\left(\tau \right) & \cos\left(\tau \right) \end{array}\right]\left[\begin{array}{c} P_{2x}\\ P_{2y}\\ P_{2z} \end{array}\right]=\left[\begin{array}{c} \cos\left(\gamma -270{}^{\circ}+\alpha \right)\\ \cos\left(\left(\gamma -270{}^{\circ}+\alpha \right)-90{}^{\circ}\right)\\ \cos\left(90{}^{\circ}\right) \end{array}\right].$$

Solving the above matrix yields

$$\left[\begin{array}{c} P_{2x}\\ P_{2y}\\ P_{2z} \end{array}\right]=\left[\begin{array}{c} \cos\left(\alpha +\gamma \right)\cos\left(\omega +180{}^{\circ}-\alpha \right)-\cos\left(\tau \right)\sin\left(\alpha +\gamma \right)\sin\left(\omega +180{}^{\circ}-\alpha \right)\\ -\cos\left(\tau \right)\cos\left(\omega +180{}^{\circ}-\alpha \right)\sin\left(\alpha +\gamma \right)-\cos\left(\alpha +\gamma \right)\sin\left(\omega +180{}^{\circ}-\alpha \right)\\ \sin\left(\alpha +\gamma \right)\sin\left(\tau \right) \end{array}\right].$$

We now solve for *N*_2_ from

$$N_{2}=P_{2}\times M_{\text{norm}}$$

to obtain

$$\left[\begin{array}{c} N_{2x}\\ N_{2y}\\ N_{2z} \end{array}\right]=\left[\begin{array}{c} -\cos^{2}\left(\tau \right)\cos\left(\omega +180{}^{\circ}-\alpha \right)\sin\left(\alpha +\gamma \right)-\cos\left(\omega +180{}^{\circ}-\alpha \right)\sin\left(\alpha +\gamma \right)\sin^{2}\left(\tau \right)-\cos\left(\alpha +\gamma \right)\cos\left(\tau \right)\sin\left(\omega +180{}^{\circ}-\alpha \right)\\ -\cos\left(\alpha +\gamma \right)\cos\left(\tau \right)\cos\left(\omega +180{}^{\circ}-\alpha \right)+\cos^{2}\left(\tau \right)\sin\left(\alpha +\gamma \right)\sin\left(\omega +180{}^{\circ}-\alpha \right)+\sin\left(\alpha +\gamma \right)\sin^{2}\left(\tau \right)\sin\left(\omega +180{}^{\circ}-\alpha \right)\\ \cos\left(\alpha +\gamma \right)\sin\left(\tau \right) \end{array}\right].$$

Finally, we solve

$$\theta_{H:S_{2}}=-\arctan\left(\frac{N_{2y}}{N_{2x}}\right)$$

and obtain

$$\theta_{H:S_{2}}=-\arctan\left(\frac{\cos\left(\tau \right)\cos\left(\alpha +\gamma \right)\cos\left(\omega +180{}^{\circ}-\alpha \right)-\sin\left(\alpha +\gamma \right)\sin\left(\omega +180{}^{\circ}-\alpha \right)}{\cos\left(\tau \right)\cos\left(\alpha +\gamma \right)\sin\left(\omega +180{}^{\circ}-\alpha \right)+\sin\left(\alpha +\gamma \right)\cos\left(\omega +180{}^{\circ}-\alpha \right)}\right).$$

### 8.3 Derivation of the Horizontal Angle Error

The horizontal angle error, *ϵ*_H_, is the difference in the horizontal angles derived above:

$$\epsilon_{H}=\theta_{H:S_{2}}-\theta_{H:S_{1}}$$

where

$$\begin{array}{c} \theta_{H:S1}=-\arctan\left(\frac{\cos\left(\tau \right)\cos\left(\gamma \right)\cos\left(\omega \right)-\sin\left(\gamma \right)\sin\left(\omega \right)}{\cos\left(\tau \right)\cos\left(\gamma \right)\sin\left(\omega \right)+\sin\left(\gamma \right)\cos\left(\omega \right)}\right)\\ \theta_{H:S_{2}}=-\arctan\left(\frac{\cos\left(\tau \right)\cos\left(\alpha +\gamma \right)\cos\left(\omega +180{}^{\circ}-\alpha \right)-\sin\left(\alpha +\gamma \right)\sin\left(\omega +180{}^{\circ}-\alpha \right)}{\cos\left(\tau \right)\cos\left(\alpha +\gamma \right)\sin\left(\omega +180{}^{\circ}-\alpha \right)+\sin\left(\alpha +\gamma \right)\cos\left(\omega +180{}^{\circ}-\alpha \right)}\right). \end{array}$$

During calibration, it is customary to align the first surface of the artifact to the reference zero of the autocollimator as close as possible. Therefore, the position angle of the mount during setup is determined by the position of the artifact on the mount:

$$\begin{array}{l} \omega =\arctan\left(\frac{\cos\left(\tau \right)\cos\left(\gamma \right)}{\sin\left(\gamma \right)}\right)\to \gamma \neq \pm \left(90N\right){}^{\circ}\\ \begin{array}{cc} \omega =90{}^{\circ}-\gamma & \;\to \gamma =\pm \left(90N\right){}^{\circ} \end{array} \end{array}$$

where *N* is a whole number from 0 to ∞. This eliminates the independent variable, *ω*.

### 8.4 Simplification of the Error Equation

The error equation is expanded in a Taylor-series around *τ* to the sixth power:

$$\begin{array}{l} \epsilon_{H}=\tau^{2}\left(\frac{\cos\left(\alpha +2y\right)\sin\left(\alpha \right)}{2}\right)\\ \;+\tau^{4}\left(\frac{\cos\left(\alpha +2\gamma \right)\sin\left(\alpha \right)}{12}+\frac{\cos\left(\alpha +4\gamma \right)\sin\left(\alpha \right)}{16}+\frac{\cos\left(3\alpha +4\gamma \right)\sin\left(\alpha \right)}{16}\right)\\ \;+\tau^{6}(\frac{17\cos\left(\alpha +2\gamma \right)\sin\left(\alpha \right)}{1440}+\frac{\cos\left(\alpha +4\gamma \right)\sin\left(\alpha \right)}{48}+\frac{\cos\left(3\alpha +4\gamma \right)\sin\left(\alpha \right)}{48}\\ \;+\frac{\cos\left(\alpha +6\gamma \right)\sin\left(\alpha \right)}{96}+\frac{\cos\left(3\alpha +6\gamma \right)\sin\left(\alpha \right)}{96}+\frac{\cos\left(5\alpha +6\gamma \right)\sin\left(\alpha \right)}{96})\\ \;+O_{3}\left(\tau \right) \end{array}$$

where *O*_3_(*τ*) represents the sum of the terms that are greater than the sixth power. The variables *ϵ*_H_ and *τ* are both expressed in radians. The above equation becomes:

$$\begin{array}{l} \epsilon_{H}\approx \tau^{2}\left(2.424\times 10^{-6}\right)\cos\left(\alpha +2\gamma \right)\sin\left(\alpha \right)\\ \;+\tau^{4}(\left(9.496\times 10^{-18}\right)\cos\left(\alpha +2\gamma \right)\sin\left(\alpha \right)\\ \;+\left(7.122\times 10^{-18}\right)\cos\left(\alpha +4\gamma \right)\sin\left(\alpha \right)\\ \;+\left(7.122\times 10^{-18}\right)\cos\left(3\alpha +4\gamma \right)\sin\left(\alpha \right))\\ \;+\tau^{6}(\left(3.162\times 10^{-29}\right)\cos\left(\alpha +2\gamma \right)\sin\left(\alpha \right)\\ \;+\left(5.580\times 10^{-29}\right)\cos\left(\alpha +4\gamma \right)\sin\left(\alpha \right)\\ \;+\left(5.580\times 10^{-29}\right)\cos\left(3\alpha +4\gamma \right)\sin\left(\alpha \right)\\ \;+\left(2.790\times 10^{-29}\right)\cos\left(\alpha +6\gamma \right)\sin\left(\alpha \right)\\ \;+\left(2.790\times 10^{-29}\right)\cos\left(3\alpha +6\gamma \right)\sin\left(\alpha \right)\\ \;+\left(2.790\times 10^{-29}\right)\cos\left(5\alpha +6\gamma \right)\sin\left(\alpha \right))+O_{4}\left(\tau \right) \end{array}$$

for *ϵ*_H_ and *τ* expressed in arcseconds. This equation is an approximation because the coefficient in front of each term is only carried out to four significant digits. In most angle calibrations, the tilt angle is less than a degree. Therefore, using only the first term in the above equations is sufficient for determining the error to a hundredth of an arcsecond.
